# Matrix Metalloproteinases and Pro-Inflammatory Cytokines in Bladder Cancer: Diagnostic and Prognostic Perspectives: Narrative Review

**DOI:** 10.3390/ijms27073218

**Published:** 2026-04-02

**Authors:** Urszula Lipka, Karolina Orywal, Monika Gudowska-Sawczuk

**Affiliations:** Department of Biochemical Diagnostics, Medical University of Bialystok, Waszyngtona 15A St., 15-269 Bialystok, Poland

**Keywords:** matrix metalloproteinases, MMP, cytokines, bladder cancer, diagnosis, prognosis, marker, inflammation

## Abstract

Bladder cancer (BC) is one of the most commonly diagnosed cancers of the genitourinary system and ranks ninth in terms of incidence worldwide. Due to the varied clinical course of the disease and the frequent tendency for tumor recurrence, diagnostic studies are being conducted to find non-invasive and prognostic markers that could be helpful in diagnosing BC. The aim of this review was to present the current state of knowledge on the role of extracellular matrix metalloproteinases (MMPs) and pro-inflammatory cytokines in the pathogenesis of bladder cancer, as well as to assess their potential as biomarkers measured in body fluids. The study reviewed the literature published in English between 2012 and 2025. The studies included clinical and experimental research analysing the expression and activity of selected metalloproteinases, including MMP-2, MMP-9, MMP-7 and MMP-1, and pro-inflammatory cytokines such as interleukins (ILs): IL-6, IL-8, IL-17, and IFN-γ in serum, urine, and tumor tissues of patients with BC, using immunochemical methods and genetic analyses. The collected data indicate that MMPs play a key role in the degradation of the extracellular matrix, facilitating invasion, angiogenesis, and metastasis. Elevated concentrations of MMP-2 and MMP-9, especially in urine, correlate with the clinical stage and histopathological malignancy of the tumor. MMP-7 may be important in the early stages of carcinogenesis, while MMP-1 shows promising potential in multi-marker panels. At the same time, chronic inflammation and increased pro-inflammatory cytokine activity play a key role in modulating the tumor microenvironment. IL-6 and IL-8 have high diagnostic value in urine tests, with IL-6 also serving as a prognostic marker. IL-17 is associated with more aggressive forms of the disease, while IFN-γ may reflect the immune response to BC treatment. There is also evidence of positive feedback between cytokines and MMPs, which intensifies the process of tumor invasion and progression. Therefore, both extracellular matrix metalloproteinases and pro-inflammatory cytokines may be promising non-invasive biomarkers of bladder cancer. Their combined assessment can increase the diagnostic and prognostic value compared to the analysis of individual parameters. However, further multicentre prospective studies are needed for clinical validation and standardisation of the methods of determination.

## 1. Introduction

Bladder cancer (BC) is the most common genitourinary malignancy and the ninth most diagnosed cancer worldwide. About 430,000 new cases are diagnosed yearly, with incidence rising mainly in developed countries [1,2]. The risk of developing bladder cancer is higher in men, with an age-standardized rate (ASR) ratio ranging from 6:1 to 2:1 depending on the latitude and place of residence [3]. The risk of the disease also increases with age. A predisposing factor for the development of cancer is exposure to carcinogens through direct contact with the skin, respiratory tract, and digestive system. The most important factors include tobacco smoke and environmental and occupational exposure [4]. About 75% of cases are non-muscle-invasive urothelial bladder cancer (NMIBC), while 25–30% are muscle-invasive (MIBC), affecting deeper bladder layers or metastasizing [5].

The initial step in the diagnosis of bladder cancer is performing a routine urinalysis with sediment examination. In patients, visible hematuria is most commonly observed, and in some cases, proteinuria and leukocyturia are also present [6]. White light cystoscopy is considered the gold standard in BC diagnosis. In addition, urine cytology, which is a non-invasive complementary test, can also be performed. When pathological tissue is detected, the patient undergoes transurethral resection of bladder tumor (TURBT). However, it should be remembered that the risk of both underestimating and overestimating the clinical assessment during the procedure is still significant. Over the years, advanced cystoscopic technologies have also been developed, such as photodynamic diagnosis (PDD) and narrow-band imaging (NBI), which can be helpful in diagnosing hard-to-detect lesions. Cystoscopy is widely used but expensive, invasive, and may cause complications. On the other hand, cytology has low sensitivity in detecting low-grade tumors. For this reason, modern medicine is still developing new diagnostic tests that may be incorporated into the management of patients with bladder cancer in the future [7,8,9].

Evidence suggests that molecular markers detected in urine, tissues, or blood may improve our understanding of the pathways involved in urothelial carcinogenesis. This could speed up the diagnosis of the disease, assist in risk profiling, and improve the prognosis of treatment outcomes [10]. Various molecular modifications are reflected in diverse cell morphology and significant tumor heterogeneity [11]. It is unlikely that there is a single marker that would fully determine the potential and dynamics of tumor development [10]. In recent years, a significant number of new biomarkers have emerged and are still being tested, many of which are related to the activation and modulation of inflammatory mechanisms that play a significant role in carcinogenesis. Based on prospective studies and meta-analyses, it can be assumed that a new generation of biomarkers will be introduced into clinical practice in the near future, which may translate into improved treatment outcomes in terms of both morbidity and mortality [8]. In this context, the aim of this publication is to review the current literature on the role of matrix metalloproteinases (MMPs) and proinflammatory cytokines in bladder cancer.

## 2. Material and Methods

This narrative review was based on a comprehensive literature search of the following electronic databases: PubMed, Scopus, and Web of Science. The search included studies published between January 2012 and March 2025. The search strategy included combinations of the following keywords: “matrix metalloproteinases”, “MMPs”, “cytokines”, “bladder cancer”, “inflammation”, “biomarkers”, “diagnosis” and “prognosis.” “AND” and “OR” were used to refine the search. Inclusion criteria included:

- original research articles and selected review articles
- studies published in English,
- studies examining the role of matrix metalloproteinases and/or proinflammatory cytokines in bladder cancer,
- studies assessing these markers in biological samples such as urine, serum, or tissue.

Exclusion criteria included:

- publications in languages other than English,
- conference abstracts without full text,
- case reports,
- studies not directly related to the topic of bladder cancer biomarkers.

The final selection of studies was based on their relevance to the topic and their contribution to understanding the diagnostic and prognostic significance of MMPs and proinflammatory cytokines in bladder cancer.

## 3. Pathogenesis

Urothelial bladder cancer (UBC) accounts for ~90% of cases; the rest include small cell, adenocarcinoma, sarcoma, and squamous cell carcinomas. Urothelial bladder cancer is characterized as a tumor originating from urothelial cells that can invade the basement membrane, lamina propria, or muscle layer [12]. Bladder cancers are classified by the TNM system (Tis–T4) and by morphological differentiation. Initially, ~60% of BCs are stage Ta papillary tumors, non-muscle-invasive and low-grade. In turn, approximately 20% of cases at the time of diagnosis are T1 stage tumors that have spread beyond the basement membrane of the epithelium but do not invade the muscle layer. They are characterized by a high degree of malignancy, similar to tumors that invade the muscle layer. In most cases, patients with NMIBC experience a recurrence of the disease, while the tumor rarely evolves into MIBC. There are two main molecular pathways in the development of bladder cancer. The first one concerns papillary NMIBC with hyperplastic urothelial changes. This pathway is characterized by the occurrence of chromosome 9 deletion and frequent FGFR3 (Fibroblast Growth Factor Receptor 3) point mutation. The second pathway concerns MIBC, and for it to be activated, changes in the bladder epithelium must first occur, including squamous urothelial dysplasia and carcinoma in situ (CIS). A characteristic feature of this pathway is the early loss of the Tp53 (tumor protein p53), Rb1 (retinoblastoma 1), and Pten (phosphatase and tensin homolog) genes, which results in the deregulation of the cell cycle, including the G1 phase [13,14]. The invasion of cancer cells is a multi-stage process in which the remodelling of the extracellular matrix (ECM) and basement membranes surrounding the primary tumor plays a key role. MMPs, which are responsible for the degradation of ECM components, play an important role in this phenomenon. Extracellular matrix metalloproteinases break down collagen, fibronectin and other structural elements, facilitating the migration of cancer cells and releasing cytokines, chemokines and growth factors stored in the matrix, which further modulate the tumor tissue environment [15]. The tumor microenvironment is a dynamic structure in which cancer cells interact with stromal cells. Cancer cells synthesise and secrete numerous mediators, such as transforming growth factor β1 (TGF-β1), fibroblast growth factor β2 (FGF-β2), interleukins (ILs): IL-1 and IL-6, and MMP-1, MMP-2 and MMP-9. These factors recruit stromal cells and modulate their phenotype, transforming them into populations that support tumor growth [16]. Cytokines bind cancer cell receptors; IL-6 activates STAT3, promoting proliferation and migration. TGF-β1 binds to TGF-βRI/II receptors, while IL-8 binds to CXCR1/CXCR2 receptors. It is believed that the binding of cytokines disrupts signalling pathways such as RAS-RAF-ERK (Rat Sarcoma-Rapidly Accelerated Fibrosarcoma-Extracellular signal-Regulated Kinase), JAK-STAT (Janus Kinase-Signal Transducer and Activator of Transcription pathway), AKT (Protein Kinase B) and SMAD, the activation of which accelerates the epithelial–mesenchymal transition in cancer cells [17]. One of the most important groups of stromal cells are cancer-associated fibroblasts (CAFs), which exhibit intense secretory activity, producing both matrix components and metalloproteinases, including MMP-1, MMP-3, MMP-7, MMP-9 and MMP-13. These enzymes enhance ECM remodelling and the release of proangiogenic factors such as VEGF (Vascular Endothelial Growth Factor). Tumor-associated macrophages (TAMs), which can take on different functional phenotypes, are also an important component of the tumor microenvironment. M1 macrophages exhibit antitumor and immunostimulatory activity, including through the secretion of interferon γ (IFN-γ) and interleukin 12. In contrast, the M2 phenotype is associated with immunosuppressive and proangiogenic effects. M2 TAMs produce interleukin 10 and increase the secretion of MMP-1, MMP-3 and MMP-14, promoting matrix degradation and tumor progression [15]. It is suggested that a more thorough understanding of the pathogenesis of bladder cancer allows for the expansion of cancer diagnosis using molecular biomarkers. This also provides a basis for prognosis, personalisation of treatment and disease monitoring [13].

## 4. Matrix Metalloproteinases (MMP)

### 4.1. Characteristics

**Figure 1:**
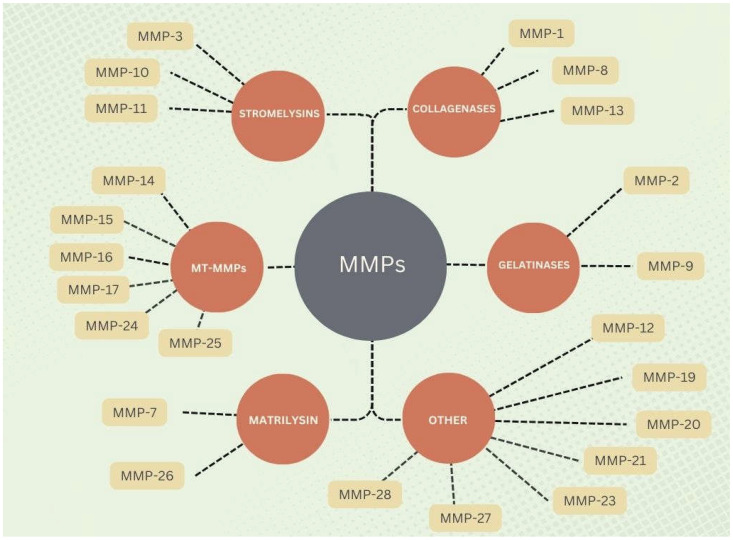
Classification of extracellular matrix metalloproteinases (MMPs).

Matrix metalloproteinases (MMPs) are proteolytic enzymes found in living organisms, where they perform various functions responsible for the regulation of physiological processes such as angiogenesis, wound healing, embryogenesis, and control of inflammatory responses [17]. MMPs form a family of 25 metalloproteinases, but only 23 of them have been identified in humans. They are endopeptidases that have been classified into six classes differing in substrate specificity: collagenases, stromelysins, gelatinases, matrilysins, membrane-type metalloproteinases (MT-MMPs), and others [18] (Figure 1).

Metalloproteinases contain up to three Ca^2+^ ions and one Zn^2+^ ion. The active center of MMPs, on the other hand, consists of a catalytic domain containing a second Zn^2+^ ion coordinated by three histidine residues. This is where protein hydrolysis takes place with the participation of a water molecule [17]. MMPs are synthesized as inactive zymogens due to propeptide interaction with catalytic Zn^2+^. Their activation occurs as a result of proteolytic cleavage. Studies have shown that MMP regulation occurs mainly at the transcriptional and post-transcriptional levels. The expression of MMP-encoding genes occurs mainly in connective tissue fibroblasts, but is also observed in monocytes, endothelial cells, macrophages, and neutrophils [18,19]. Tissue inhibitors of metalloproteinases (TIMPs) are responsible for the precise regulation of MMP activity. When a pathological condition caused by diseases such as cancer, arthritis, sepsis, chronic obstructive pulmonary disease (COPD), or neurological disorders occurs, the balance in the system is disrupted, resulting in increased activity of matrix metalloproteinases. This can lead to many adverse events, including matrix weakening, fibrosis, and tissue degradation. In recent years, scientists have taken a keen interest in cancer research, linking MMPs to disease progression. This applies to cancers whose risk increases significantly after the age of 50, including bladder cancer [17,20].

### 4.2. Mechanism of Action of MMP on the Development of BC Carcinogenesis

In recent years, cancer processes have become the focus of research, and MMPs have been recognized as one of the key factors in the development of carcinogenesis. They promote tumor growth, invasion, angiogenesis, and metastasis. According to the available scientific literature, matrix metalloproteinases appear at every stage of carcinogenesis: initiation, promotion, progression, and metastasis. In the case of bladder cancer, studies primarily focus on MMP-2 and MMP-9 [15].

Matrix metalloproteinase 2 (MMP-2), also known as gelatinase A or neutrophil gelatinase, is a protease with a molecular weight of 66 kDa. It breaks down gelatin, laminin, fibronectin, aggrecan, elastin, proteoglycans, and collagens of types I, II, III, IV, V, VII, X, and XI. It degrades ECM and basement membrane, enabling tumor cell migration. Cancer cells have their own ability to produce MMP-2 or initiate its production in neighboring cells. This leads to the creation of a microenvironment that promotes tumor growth and invasion [21,22].

Another proteinase with proteolytic activity against collagen IV is matrix metalloproteinase 9 (MMP-9), which belongs to the gelatinase family [23]. Its molecular weight is 86 kDa. It also influences the structural modification of the ECM, and its increased expression results in the development of carcinogenesis [15]. MMP-9 has been identified as a prognostic indicator for immune cells infiltrating the tumor and for prognosis. Based on the studies conducted, it was concluded that neutrophils infiltrating tumor tissue may play a key role as a source of MMP-9 in the tumor microenvironment. In addition, epithelial–mesenchymal transition induced by increased MMP-9 concentration enhances the invasion of bladder cancer cells [24].

The available literature provides information on the impact of matrix metalloproteinase 1 (MMP-1) on carcinogenesis. MMP-1 breaks down type I, II, and III interstitial collagen, facilitating the invasion of cancer cells through the extracellular matrix and their movement within the tissue stroma [25]. In addition, MMP-1 degrades other substrates, including gelatin, laminin, complement component C1, interleukin-1 (IL-1), tumor necrosis factor (TNF), and insulin-like growth factor-binding proteins. Previous studies indicate MMP-2 and MMP-9 may be activated by MMP-1 [22].

Matrix metalloproteinase 7 (MMP-7), which belongs to the matrilysin family, also plays a role in the development of bladder cancer. It has the ability to break down ECM components and molecules that appear on the surface of cells [26]. Its molecular weight is 28 kDa. MMP-7 is called the smallest member of the MMP family due to the absence of an additional hemopexin-like domain, which is responsible for substrate specificity [22]. Researchers suggest that the highest activity of MMP-7 can be observed mainly in the early stages of carcinogenesis. Matrix metalloproteinase 7 is multifaceted: it can inhibit apoptosis and intensify osteolysis, resulting in bone metastases [23]. It is suggested that MMP-7 may be an independent prognostic factor in patients with locally advanced and metastatic BC [27].

It is believed that increased activity of matrix metalloproteinase 14 (MMP-14) also influences the carcinogenic processes of BC. MMP-14 is also known as membrane metalloproteinase type 1 (MT1-MMP). It is found in many cell types and plays an important role in physiological processes. It affects angiogenesis through the degradation of type I, II, and III collagen. It has the ability to stimulate pro-MMP-2, thereby enhancing local tumor invasion and metastasis. High concentrations of MMP-14 may be associated with advanced tumor malignancy, aggressiveness, or poor prognosis for survival [22,28].

### 4.3. Use of MMPs as Biomarkers in Body Fluids

Fouad H et al. showed increased MMP-2/TIMP-2 and MMP-9/TIMP-2 ratios in bladder cancer patients vs. controls. The analysis was performed using the Western blot method. These results suggest that increased concentrations of proteolytic enzymes compared to their inhibitors lead to the degradation of ECM, which promotes tumor growth [29]. In turn, in studies conducted by Ricci et al. using the ELISA method, the concentrations of MMP-2 and MMP-9 were assessed in urine and serum samples from BC patients and healthy individuals. MMPs were absent in urine of healthy individuals, indicating low physiological activity. On the other hand, MMP-2 and MMP-9 were detected in the majority of patients with bladder cancer. Matrix metalloproteinase 9 was detected in approximately 61% of patients, with a median concentration in urine of 1.30 ng/mL, while matrix metalloproteinase 2 was found in 63% of patients, with a median concentration in urine of 1.27 ng/mL. MMP-2 and MMP-9 levels may reflect tumor malignancy grade. The lowest concentrations of both metalloproteinases were found in tumors with a low degree of malignancy (G1) and superficial lesions (Ta), while their concentrations increased in higher categories of local advancement (T1–T2) and in tumors with a higher degree of malignancy (G3). The researchers also showed that the determination of these metalloproteinases in urine has greater diagnostic value than in serum, as the differences in MMP-2 and MMP-9 concentrations in the blood between patients and the control group were less noticeable [30]. Similar results were obtained by Li Chen and Junhui He, who evaluated the diagnostic usefulness of MMP-9 and nuclear matrix protein 22 (NMP22) in blood serum in patients with bladder cancer using the ELISA method. The analysis showed that MMP-9 and NMP22 concentrations were statistically significantly higher in BC patients compared to the control group (*p* < 0.001). In addition, the studies showed a correlation with local tumor progression, as the concentrations of both MMP-9 and NMP22 were highest in patients in stage T, while significantly lower concentrations were observed in stages N and M [31]. Studies conducted by Mlynarczyk G et al. focused on the analysis of the expression and activity of MMP-7 and MMP-26 in patients with bladder cancer. The study group was divided into two subgroups: patients with low-grade and high-grade urothelial carcinoma. The results showed that the activity and expression of MMP-7 significantly increased in pathological tissues compared to normal tissues. These differences are also observed in body fluids, which may indirectly indicate increased infiltration from the tumor area into the circulatory system. In addition, significantly higher specific activity of MMP-7 compared to MMP-26 has been demonstrated, indicating the leading role of matrix metalloproteinase 7 in ECM remodeling. These processes promote cancer cell invasion, confirming the key role of MMP-7 in early carcinogenesis. MMP-26 shows no significant activity in bladder cancer, suggesting a minor role in its development [26]. Vanarsa et al. identified MMP-1 (AUC ≈ 0.89) as one of the most accurate urinary biomarkers for bladder cancer, indicating its diagnostic potential, especially in multi-marker tests [32].

## 5. Proinflammatory Cytokines

Cytokines are a group of signaling proteins that stimulate complex mechanisms involved in immune and inflammatory responses. They are responsible for stimulating phagocytic macrophages, are involved in the differentiation of T helper (Th) lymphocytes into Th1 and Th2 subtypes, and regulate the process of antibody class switching in B lymphocytes [33]. Cytokines can exert different and, in some cases, opposing biological effects depending on the temporal context, while participating in a feedback mechanism that controls the intensity of their expression. The conditions prevailing in the cellular microenvironment determine the level of cytokine expression, indicating the existence of complex mechanisms that control the effectiveness of the immune response and prevent its excessive and prolonged activation [34]. Cytokines can be secreted by various cells in the body, such as macrophages, CD4+ lymphocytes, Th1 cells, and dendritic cells. The cytokine family is divided into interleukins (IL), interferons (IFN), tumor necrosis factors (TNF), granulocyte-macrophage colony-stimulating factors (GM-CSF), and chemokines [33]. The main pro-inflammatory cytokines are IL-1, IL-6, IL-8, and TNF-α. They transmit signals through type I cytokine receptors (CCR1), which differ structurally from other classes of cytokine receptors [33,35]. The main biological functions of cytokines include their effect on elevated body temperature. IL-1 is one of the first pro-inflammatory mediators produced during fever, which induces the production of prostaglandins in the hypothalamus. Cytokines are also responsible for stimulating leukocytes. In addition, evidence suggests that IL-1, IL-6, and TNF increase the synthesis of acute phase proteins in the liver [36].

### 5.1. The Role of Proinflammatory Cytokines in the Development of Bladder Cancer

The process of transformation of normal transitional epithelium into bladder cancer consists of many determinants. A number of scientific studies have proven that chronic inflammation is involved in the activation and development of invasive and metastatic tumors [37]. Cytokines are mediators involved in the progression mechanisms of many malignant tumors and the regulation of their microenvironment, including bladder cancer, but the full characteristics of the cytokine profile in this cancer remain unclear [38]. Based on current data from the literature, research indicates the key pro-inflammatory cytokines playing an important role in BC are IL-6, IL-8, and IL-1 [39].

Interleukin 6 is a potential biomarker for bladder cancer, as it can induce tumorigenesis and influence tumor development and prognosis [40]. IL-6 stimulates cancer stem cell properties and binds to the IL-6 receptor (IL6R), activating the IL-6/STAT3 pathway [41]. This signaling promotes cell survival, proliferation, angiogenesis, invasion, and metastasis, upregulates anti-apoptotic genes like Mcl-1 and cyclin A, and increases MMP2 and MMP9 production, enhancing cancer cell invasiveness [42]. Another cytokine, interleukin 8, also known as CXCL8, has been shown to contribute to the development of bladder cancer. IL-8 binds to CXCR1 and CXCR2 receptors on local cells, thereby initiating neutrophil chemotaxis. It has been suggested that interleukin 8 may show significantly increased expression in invasive and highly malignant urothelial bladder cancer tumors, as it may promote tumor progression through various mechanisms. The CXCR2 receptor for IL-8 promotes the formation of new blood vessels in the tumor and facilitates the influx of leukocytes into its microenvironment. The recruitment of neutrophils is particularly disadvantageous, as tumor-associated neutrophils can induce epithelial–mesenchymal transition in cancer cells, which promotes metastasis. The presence of IL-8 also correlates with the size and stage of the tumor. In addition, CXCL8 stimulates the expression of genes responsible for cell proliferation and reduces the activity of tumor suppressor genes, acting through the mitogen-activated protein kinase (MAPK) and Janus kinase 2 (JAK2) signaling pathways, as well as focal adhesion kinase (FAK). Therefore, it has been proven that interleukin 8 participates in the activation of the carcinogenesis process, creates a favorable environment for cell migration, promotes tumor growth, and is responsible for inhibiting apoptosis [33,38].

The available literature describes the important role of interleukin 1 and its antagonist (IL-1RA) in inflammation and BC development. Increased expression of IL-1β in invasive bladder cancer is associated with blood and lymphatic vessel invasion, tumor aggressiveness, and poorer patient prognosis. In contrast, reduced IL-1RA levels lead to greater invasiveness and facilitated migration of BC cells [39].

Scientific studies have also been conducted which have shown that the activity of IL-17 as a factor promoting neoplasia plays an important role in the onset and development of bladder cancer. Interleukin 17 is involved in many stages of tumor pathophysiology, including carcinogenesis, tumor cell proliferation, angiogenesis, and metastasis, as well as the development of tumor resistance to immune mechanisms and chemotherapy [43].

Tumor-infiltrating lymphocytes produce interferon gamma (IFN-γ), which plays an important role as a pro-inflammatory cytokine in bladder cancer. IFN-γ has been shown to be responsible for modulating the tumor microenvironment. Under physiological conditions, interferon gamma induces phosphorylation of signal transducer and activator of transcription 1 (STAT1) in tumor cells, initiating interferon-dependent signaling pathways, which results in enhanced antigen presentation and influences the more effective activity of cytotoxic CD8+ T lymphocytes by regulating the expression of chemokine ligands with the C-X-C motif (CXCL), interferon-stimulated genes (ISGs), as well as genes from the human leukocyte antigen (HLA) family [44,45].

### 5.2. Diagnostic Use of Proinflammatory Cytokines as Biomarkers in Body Fluids

In a study conducted by Vanden Bussche et al., IL-6 and IL-8 concentrations in urine were compared in patients with urothelial bladder cancer and a control group of healthy individuals. Immunoenzymatic methods were used to perform the measurements. It was shown that individuals with urothelial bladder cancer had statistically significantly higher concentrations of both cytokines in their urine compared to the control group. In addition, the combination of IL-6 and IL-8 showed high sensitivity (90%) and specificity (81.25%), which may be of clinical significance, especially in highly malignant tumors. The results obtained in the above study suggest that both IL-6 and IL-8 may be associated with the presence of BC and pathological processes occurring in the bladder wall [38]. The study by Kumari et al. analyzed 27 cytokines in the serum and urine of patients with bladder cancer to assess their relationship with tumor invasiveness and the risk of recurrence. Among them, particular attention was paid to IL-6 and IL-8, which showed significant differences between patients and healthy individuals. ELISA immunoenzymatic assays were used to perform the measurements. Statistically significantly higher serum IL-6 concentrations were observed in patients with disease recurrence. Kaplan–Meier analysis showed that high IL-6 concentrations were associated with shorter recurrence-free survival (RFS), which may reflect the role of interleukin-6 as a prognostic marker of BC recurrence. In contrast, IL-8 concentrations, measured mainly in urine, were also elevated in patients, especially those with recurrence, but no direct correlation with shorter RFS was demonstrated. These data indicate that both cytokines may play a supporting role in assessing the risk of recurrence and aggressiveness of bladder cancer, with serum IL-6 showing greater prognostic potential and urinary IL-8 reflecting the local inflammatory response in the tumor microenvironment [46]. Engelmann et al. conducted a study involving 179 patients with bladder cancer in whom serum IL-6 concentrations were measured by ELISA prior to planned radical cystectomy. In the analyzed patients, the median IL-6 concentration in the blood was 5.4 pg/mL. It was shown that elevated IL-6 concentrations in the blood were a significant independent factor for poorer overall survival (OS) and bladder cancer-specific survival (CSS) (HR ~1.95; HR ~2.31, respectively). In addition, patients with larger tumor sizes and lymph node metastases had statistically higher IL-6 concentrations in the blood. It can therefore be suggested that in a given patient population, higher serum interleukin-6 concentrations are associated with a higher risk of overall mortality as well as BC-specific mortality and aggressive clinical features of the tumor. Therefore, IL-6 may be a promising prognostic biomarker in bladder cancer [40]. In a study by Mousa et al., serum and urine IL-17 concentrations were compared in 50 patients with bladder cancer and 96 healthy individuals using the ELISA method. It was shown that both in urine and serum, the concentration of interleukin-17 was statistically significantly higher in patients (*p* < 0.05). Furthermore, IL-17 concentrations were particularly high in patients with MIBC. These data suggest that elevated interleukin-17 concentrations are associated with the occurrence of bladder cancer and correlate with the occurrence of more aggressive forms of the disease, which may find clinical application as a prognostic biomarker or a tool for monitoring the disease [43]. In a study by El-Gazzar et al., the significance of immune markers, including interferon gamma, in predicting response to BCG (Bacillus Calmette-Guerin) therapy in high-risk NMIBC patients was analyzed. The measurement was performed in urine using the ELISA method. The researchers showed that lower concentrations of IFN-γ in urine prior to treatment were significantly associated with a higher risk of disease recurrence (*p* < 0.001). These results may indicate that interferon gamma may reflect the activation of a local inflammatory response against the tumor. In the multivariate analysis, the effect of IFN-γ did not remain statistically significant, which may have been due to correlation with other markers studied. These results indicate that IFN-γ may be used as a non-specific, prognostic biomarker of immune response in NMIBC after BCG treatment [47]. A summary of selected pro-inflammatory cytokines and their diagnostic significance in body fluids is presented in Table 1.

## 6. Relationship Between MMP Expression and Proinflammatory Response in BC

**Figure 2:**
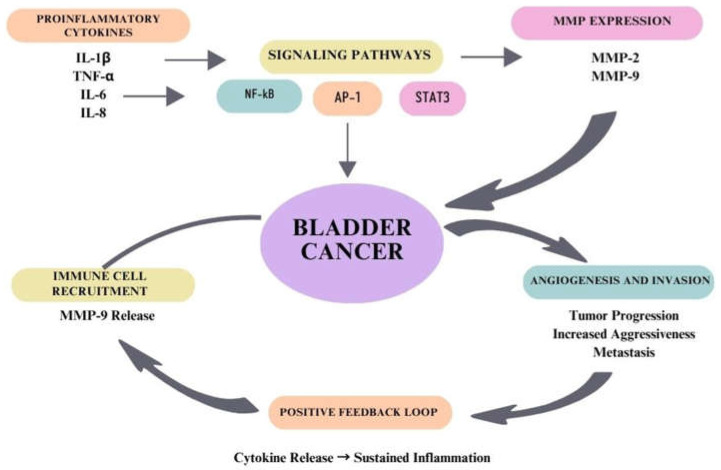
The interaction between proinflammatory cytokines, MMPs, and tumor progression in bladder cancer.

It has been proven that the regulation of the expression and activity of extracellular matrix metalloproteinases in bladder cancer can be stimulated by proinflammatory cytokines. The main role in these processes is played by cytokines such as IL-1β, TNF-α, IL-8, and IL-6, which have the ability to directly induce MMP expression, especially MMP-2 and MMP-9. The regulation of cellular activity by cytokines leads to the activation of intracellular signaling pathways, mainly NF-kB, AP-1, and STAT3. As a result of the stimulation of these factors, there is increased transcription of MMP-encoding genes, which contributes to increased proteolytic activity in the tissue microenvironment. This is followed by increased degradation of type IV collagen and other components of the basement membrane. It has been shown that IL-8 indirectly leads to increased MMP-9 activity by stimulating inflammatory cells to secrete it. The presence of a larger number of neutrophils contributes to the release of MMP-9 from cell granules, which further strengthens the enzymatic tissue environment. Similarly, it has been proven that TNF-α and IL-1β also influence the activation of MMP-9, which contributes to increased tumor aggressiveness and a tendency to metastasize. There is a positive feedback mechanism between cytokines and MMPs. Proteolysis of the extracellular matrix by MMPs promotes the release of associated cytokines and growth factors, leading to a continuous intensification of the inflammatory response and maintaining high expression of pro-inflammatory cytokines [48,49]. The interaction between proinflammatory cytokines, MMPs, and tumor progression in bladder cancer is presented in Figure 2.

The current scientific literature does not include many studies focusing on the simultaneous assessment of MMPs and proinflammatory cytokines in body fluids in people with bladder cancer. One of the most interesting analyses was conducted in a study by Urquidi et al., which evaluated the usefulness of selected inflammatory and proteolytic markers in urine as potential biomarkers of bladder cancer, with particular emphasis on interleukin-8 and extracellular matrix metalloproteinase 9. The concentrations of these proteins were determined using the ELISA method. The study included 127 people, including 64 with active bladder cancer and 63 without a cancer diagnosis. The median IL-8 concentration in urine in the study group was 128.43 pg/mL, while the MMP-9 concentration was 0.95 ng/mL. The median concentration of both parameters in urine in the control group was 0 pg/mL. Both differences were statistically significant (*p* < 0.0001). This means that both IL-8 and MMP-9 are associated with the presence of cancer. Among the biomarkers evaluated, IL-8 showed the highest area under the ROC curve (AUROC = 0.79), high specificity (97%), and positive predictive value (95%). In contrast, the ROC analysis of MMP-9 showed moderate diagnostic value for this marker (AUROC = 0.75), but unlike IL-8, MMP-9 did not show independent predictive value after taking into account clinical covariates. This difference may be due to the fact that MMP-9 expression is associated not only with neoplastic processes, but also with tissue remodeling and nonspecific immune response. The results obtained indicate that the determination of IL-8 in urine may reflect the degree of malignancy of bladder cancer, while MMP-9 is a useful marker to aid in the diagnosis of BC. At the same time, elevated concentrations of both markers suggest a mutual relationship between IL-8 and MMP-9, in which IL-8 may stimulate the expression of MMP-9, intensifying the process of angiogenesis and tumor invasion [50]. In addition to analyzing the relationship between MMP expression and pro-inflammatory response in body fluids, genetic studies were also conducted to describe this relationship. The analysis by Reis et al. evaluated the expression of matrix metalloproteinases and IL-8 in bladder cancer tissues to determine their prognostic significance. The study used tumor samples from 40 patients with BC and control tissues, while gene expression levels were determined by quantitative real-time PCR. In the study group, the level of MMP-9 expression in tissues was significantly higher than in the control group, and these results correlated with a higher degree of malignancy and greater tumor invasiveness. A similar relationship was observed for IL-8. In addition, higher expression of both MMP-9 and IL-8 was found in patients with disease recurrence. These results also indicate a close relationship between MMP activity and pro-inflammatory response in the tumor microenvironment [51]. In a study by Kim et al., gene expression was analyzed in bladder cancer tissues at different stages of advancement, showing significant differences between NMIBC and MIBC tumors. The analysis showed that the progression of bladder cancer to a muscle-invasive form is associated with the activation of immune and inflammatory responses. Functional studies conducted on BC cell lines showed that IL-5, IL-20, and IL-28A significantly increase the migration and invasiveness of cancer cells without affecting their proliferation rate. This effect was associated with increased expression of extracellular matrix metalloproteinases MMP-2 and MMP-9, which play an important role in ECM degradation and promote tissue invasion [52].

The key biomarkers discussed in this review are summarized in Table 2.

## 7. Research Limitations and Implications

Studies evaluating the usefulness of individual biomarkers in the diagnosis of bladder cancer still lack a standardized framework that would allow more proteins to be introduced into clinical practice. In recent years, decisions have been made to implement urine-based tests aimed at identifying individual non-invasive markers or their patterns. The usefulness of serum-based biomarker studies has also been demonstrated, as they may be important for predicting the course of BC or informing therapeutic decisions [53]. However, despite the encouraging prospects of the results obtained, which would contribute to supporting clinical decision-making in BC, these studies are at an early stage of development [10]. There are many limiting factors that may affect the results, including confounding factors such as urinary tract infections, the inclusion of a relatively small number of patients in the studies, or the need for larger, prospective studies to confirm clinical utility and control biological and technical variability [38]. It has also been suggested that the measurement of certain proteins as biomarkers may be more valuable when combined with other markers in a single panel [50]. Implications may also include early-stage testing, costs, and heterogeneous methods of results [54]. Therefore, larger prospective cohort studies will still be necessary to identify new, robust biomarkers for bladder cancer [10].

In summary, these biomarkers are unlikely to replace current diagnostic methods such as cystoscopy, which remains the gold standard for bladder cancer detection. However, they may serve as valuable complementary tools, particularly in non-invasive testing using urine or blood samples. This approach could improve early detection, patient monitoring, and risk stratification. In the future, combining multiple biomarkers into panels may further enhance diagnostic accuracy and clinical utility. Integration with existing clinical parameters and imaging techniques may also contribute to more personalized management of patients with bladder cancer.

## 8. Critical Literature Assessment

Numerous studies have examined the role of matrix metalloproteinases and proinflammatory cytokines in bladder cancer, but the reported results are not always clear and consistent. These discrepancies may be due to multiple factors. First, studies often include populations of patients with varying stages of bladder cancer progression and malignancy. Furthermore, patients with comorbidities are not always excluded, which may influence MMP and cytokine levels, thus impacting the assessment of their diagnostic value. Another contributing factor is the variability in study group sizes, which may reduce statistical power and result in inconsistent findings. Furthermore, not all assays were performed using the same diagnostic method and in the same sample. Different methods are also associated with different cutoff points. These inconsistencies may contribute to the variability of reported biomarker levels and limit the direct comparability of studies. Taking the above into account, these factors highlight the need for appropriately designed prospective studies with standardized methods to better define the clinical utility of MMPs and proinflammatory cytokines in bladder cancer.

## 9. Summary and Conclusions

This review analyzed scientific studies published in English between 2012 and 2025, which aimed to demonstrate the role of potential biomarkers useful in the diagnosis of bladder cancer. The selected publications focused on the most frequently reported extracellular matrix metalloproteinases and proinflammatory cytokines associated with BC. This approach enabled the comparison of protein expression results and facilitated the evaluation of their diagnostic relevance in carcinogenesis as well as across different stages of the disease. Additionally, publications reporting studies that demonstrated interactions between MMPs and proinflammatory cytokines in bladder cancer were included to better elucidate their combined diagnostic value. The studies were mainly based on the determination of parameters in body fluids using immunochemical methods, but two scientific articles that used genetic testing were also referenced. The available evidence supports the clinical relevance of assessing both extracellular matrix metalloproteinases and proinflammatory cytokines in patients with bladder cancer, particularly using non-invasive samples such as urine. Rather than considering individual biomarkers in isolation, the data suggest that their combined evaluation more accurately reflects key biological processes underlying tumor development, including extracellular matrix degradation, inflammation, and tumor progression. Matrix metalloproteinases play an important role in the pathogenesis of bladder cancer by intensifying ECM degradation and facilitating the invasion of cancer cells. The concentrations of MMP-2 and MMP-9 correlate with the clinical stage and histopathological malignancy of the tumor [30]. Proinflammatory cytokines, particularly IL-6 and IL-8, demonstrate high sensitivity and diagnostic specificity, highlighting their potential utility in non-invasive diagnosis, especially in high-grade disease [38]. Elevated levels of selected MMPs and cytokines are consistently associated with the presence of bladder cancer, indicating their potential utility in both diagnosis and risk stratification. Importantly, urine-based measurements appear to offer greater diagnostic value than serum analysis, highlighting the promise of non-invasive biomarker-based approaches. There is a close relationship between the inflammatory response and matrix metalloproteinase activity in bladder cancer, confirming the interaction between proinflammatory cytokines and proteolytic enzymes in the tumor microenvironment. However, despite these encouraging findings, the current evidence is limited by variability in study design, lack of standardized methodologies, and relatively small patient cohorts. These limitations hinder the direct translation of individual biomarkers into clinical practice. Therefore, future research should focus on the development and validation of standardized, multi-marker panels integrating MMPs and proinflammatory cytokines, which may ultimately improve diagnostic accuracy and support clinical decision-making in bladder cancer management [50,51,52].

## Figures and Tables

**Table 1:** Diagnostic significance of proinflammatory cytokines in body fluids.

Cytokine	Body Fluid	Significance	References
IL-6	urine, serum	Potential diagnostic and prognostic biomarker	[38,40,46]
IL-8	urine	Marker distinguishing cancer from controls and reflecting the local immune response in the tumor microenvironment	[38,46]
IL-17	urine, serum	Potential prognostic marker	[43]
IFN-γ	urine	Non-specific prognostic marker of immune response after BCG therapy	[47]

**Table 2:** Summary of selected biomarkers in bladder cancer.

Biomarker	Clinical Relevance	Diagnostic Value	Prognostic Value	Tested Sample
MMP-2	ECM degradation, tumor invasion	moderate	associated with tumor progression	urine, tissue
MMP-9	metastasis, tumor progression	high (urine)	poor prognosis, recurrence risk	tissue, urine, serum
IL-6	tumor growth, inflammation	moderate	correlates with advanced stage	urine, serum
IL-8	tumor progression angiogenesis	high (urine biomarker)	associated with recurrence	urine, serum
IL-17	immune modulation	limited	potential role in tumor progression	tissue, serum
IFN-γ	immune response regulation	limited	context-dependent (immune activity)	tissue, serum

## Data Availability

No new data were created or analyzed in this study. Data sharing is not applicable to this article.
